# Supplementing Diets with *Agriophyllum squarrosum* Reduced Blood Lipids, Enhanced Immunity and Anti-Inflammatory Capacities, and Mediated Lipid Metabolism in Tan Lambs

**DOI:** 10.3390/ani12243486

**Published:** 2022-12-09

**Authors:** Dan Jiao, Yanping Liang, Shanshan Zhou, Xiukun Wu, Abraham Allan Degen, Jonathan Hickford, Huitong Zhou, Haitao Cong, Xinxin Shi, Xiaofei Ma, Guo Yang

**Affiliations:** 1 Key Laboratory of Stress Physiology and Ecology in Cold and Arid Regions, Department of Ecology and Agriculture Research, Northwest Institute of Eco-Environment and Resources, Chinese Academy of Sciences, Gansu Province, Lanzhou 730000, China; 2 Faculty of Environmental Science and Engineering, Shanxi Institute of Science and Technology, Jincheng 048000, China; 3 Desert Animal Adaptations and Husbandry, Wyler Department of Dryland Agriculture, Blaustein Institutes for Desert Research, Ben-Gurion University of Negev, Beer Sheva l8410500, Israel; 4 Gene-Marker Laboratory, Department of Agricultural Sciences, Lincoln University, Lincoln 7647, New Zealand; 5 Shandong Huakun Rural Revitalization Institute Co., Ltd., Jinan 250000, China; 6 Dongying Modern Animal Husbandry Development Service Center, Dongying 250100, China

**Keywords:** adipose tissue, *Agriophyllum squarrosum*, amaranthaceae, lipidomics, Tan sheep, transcriptome

## Abstract

**Simple Summary:**

*Agriophyllum squarrosum* (sand rice) is an annual psammophyte that has been used in traditional Chinese medicine for many years. Its medicinal properties have been studied in mice and human, but the nutritional value and metabolic function of the above-ground parts as a ruminant feed ingredient have not been reported. Here, we find that supplementing lamb diets with *A. squarrosum* reduced blood lipids, enhanced immunity and anti-inflammatory capacities, and mediated lipid metabolism in adipose tissue and adipocytes of Tan lambs. These findings have provided strong evidence that *A. squarrosum* has the potential to be used as a beneficial dietary supplement for ruminants.

**Abstract:**

*Agriophyllum squarrosum* (sand rice), a widespread desert plant, possesses anti-hyperglycemic and anti-inflammatory properties, and has been used in traditional Chinese medicine for many years. However, its effects on ruminants are unknown. To fill this gap, we examined the effects of *A. squarrosum* on the immune and anti-inflammatory responses of lambs. A total of 23, 6-month-old Tan ewe-lambs (27.6 ± 0.47 kg) were divided into four groups and offered a basic diet (C—control), or a diet that contained 10%, 20%, or 30% *A. squarrosum*, on a dry matter basis, for 128 days. Serum concentrations of total cholesterol were lower (*p* = 0.004) in the 30% supplemented lambs than controls, while concentrations of high-density lipoprotein cholesterol were lower (*p* = 0.006) in the 10% and 20%, but not in 30% supplemented lambs than controls. Serum-cortisol concentrations were lower (*p* = 0.012) in the 30% supplemented lambs and free fatty acid concentrations were higher in the 10% and 20% supplemented lambs than in control lambs (*p* < 0.001). Supplementation with *A. squarrosum* decreased (*p* < 0.05) the area of adipocytes in subcutaneous adipose tissue, but there was no difference between the 20% and 30% diets. Conversely, the area in visceral adipose tissue (VAT) increased (*p* < 0.05), especially for the 10% and 20% supplemented diets. Supplementation with *A. squarrosum* also enriched immune and anti-inflammatory related and lipid and glucose-metabolic pathways and associated differentially expressed gene expressions in adipose tissue. A total of 10 differential triacylglycerol, 34 differential phosphatidylcholines and seven differential phosphatidylethanolamines decreased in the diet with 30% supplementation, when compared to the other diets. Finally, adipocyte-differentiation genes, and immune and inflammatory response-related gene expression levels decreased in lamb adipocytes cultured with an aqueous *A. squarrosum* extract. In conclusion, supplementing lamb diets with *A. squarrosum* reduced blood lipids, enhanced immunity and anti-inflammatory capacities, and mediated lipid metabolism in adipose tissue and adipocytes of Tan lambs. A level of approximately 10% is recommended, but further research is required to determine the precise optimal level.

## 1. Introduction

*Agriophyllum squarrosum* is an annual psammophyte, well adapted to sandy desert regions, and is distributed mainly in the arid and semi-arid areas of Central Asia. This plant has been named “sand rice” in northern China, as its seeds are eaten by humans during periods of food shortage [1,2]. The seed has a balanced composition of nutrients, with relatively high lipid (10.6% DM) and protein (24.2% DM) contents, and a full range of human-essential amino acids [3,4]. This resilient plant is also an important fodder-forage for cattle in Kazakhstan [5].

*Agriophyllum squarrosum* has been used by Mongolian doctors in China as a cure for diabetes and as a detoxifying agent [1,2]. Studies reported that it lowered blood sugar concentration, improved liver and kidney functions of rats [6], was involved in the inhibition of NF-KB protein, and decreased the concentration of inflammatory cytokines [7]. Oligo saccharides from *A. squarrosum* improved insulin resistance and reduced blood glucose in Goto-Kakizaki (GK) rats [8].

Adipose tissue is a complex organ with whole-body immunometabolic functions. It generates and sustains a strong inflammatory response to external stimuli by adipocytes and parts of the vascular matrix [9]. An inflammatory response causes adipocyte-stress responses, including hypertrophy, hypoxia, and endoplasmic-reticulum stress, which alters adipokine secretion and a macrophage-like phenotype in adipocytes [10]. An increase in adipocyte size alters the ability of adipocyte membranes to adapt to adipose tissue expansion, potentially leading to higher susceptibility to inflammation [11]. Increased levels of circulating insulin in peripheral blood may be related to increased levels in immune factor CD4 in obese children [12]. Whole-body inflammation induced by intravenous lipopolysaccharide (LPS) increases lipolysis in adipose tissue through immune signaling, such as through TLR4 in adipocytes, thereby releasing free-fatty acids (FFAs) into the blood [13]. These studies suggest that as an immune organ, adipose tissue controls a close relationship between blood, lipids, and adipocyte metabolism in the immune system.

Tan sheep, a breed well adapted to arid conditions and cold, originated in Mongolia, and, today, are distributed widely in arid and semi-arid regions of China. Given the role that *A. squarrosum* is reputed to play in anti-inflammatory activity, and its important immunometabolic functions of adipose tissue, we hypothesized that *A. squarrosum* would have beneficial effects on the immune system and lipid metabolism via adipose tissue in sheep. To test this hypothesis, we examined the effect of different levels of supplementary *A. squarrosum* on serum metabolites, adipose tissue, and cultured adipocytes. This is the first study on regulatory mechanisms of *A. squarrosum* in mammalian adipose tissue.

## 2. Materials and Methods

### 2.1. Experimental Design, Sample Collection and Histology

All procedures on the lambs were approved by the Animal Care Committee of the Northwestern Institute of Eco-Environment Resources, Chinese Academy of Sciences (protocol number: CAS201810082).

The study was undertaken at the Gansu Gaolan Field Scientific Observation and Research Station for Agricultural Ecosystems, Lanzhou, China (36°14′16 N, 103°47′59 E). A total of 23, 6-month-old Tan ewe-lambs of similar weight (27.6 ± 0.47 kg) were purchased from a feedlot in the Ningxia region. Tan ewes generally give birth to a single lamb, and, in the present study, were all single lambs. The lambs were vaccinated as required against diseases and injected subcutaneously with ivermectin (0.02 mL/kg weight; Harbin Zhongda Veterinary Medicine Co., Ltd., Harbin, China) to protect against parasites. They were maintained in individual metabolic pens (0.5 × 1.25 × 1.65 cm) with free access to feed pellets and water [14]. The pellets were composed mainly of corn kernels and lesser amounts of corn straw and alfalfa hay. The lambs were fed twice daily, at 07:00 and 17:00, and after 10 days of preliminary feeding, were divided into 4 groups in a completely randomized design. The above-ground parts of mature *A. squarrosum* were ground into fine segments, then were included in the pellets at different levels to substitute for corn stover and alfalfa hay. One group served as control with no *A. squarrosum* (C; n = 5), while the other three groups were offered pellets that contained either 10% (hereafter called 10%; n = 6), 20% (n = 6), or 30% (n = 6) *A. squarrosum* on a dry-matter basis (Table 1) [14]. The number of ewe-lambs in each treatment was based on the effect size index (d-value), which was calculated using estimated standard deviations of the means of measured variables from previous similar studies. A sample size of 6 per group resulted in a d-value close to 0.5, which, according to Sullivan and Feinn, is a medium and acceptable effect size [15]. The sheep were fed their diets for 128 days and feed remains for each sheep were collected before 7:00 each day to determine daily feed intake.

The sheep were weighed prior to feeding on days 0, 30, 60, 90, and 128 [14]. On day 129, 5 mL of jugular-vein blood were collected after 12 h of fasting and serum was collected after centrifugation at 3000 *g* at room temperature for 10 min. All the lambs were then slaughtered humanely and subcutaneous adipose tissue, including dorsal subcutaneous adipose tissue and abdominal subcutaneous adipose tissue, and visceral adipose tissue, including peri-aortic adipose tissue and peri-renal adipose tissue, were collected in 1 cm^3^ blocks. The tissues were rinsed with physiological saline and fixed in 4% paraformaldehyde solution, dehydrated in ethanol with incremental concentrations from 75% to 100%, embedded in paraffin, cut into 4 μm sections using a microtome, and then stained with hematoxylin and eosin (H&E). The sections were observed under a microscope (Leica DM750, Wetzlar, Germany), and images were analyzed using the ImageJ (version 1.53a, National Institutes of Health, Bethesda, MD, USA) software. The dorsal subcutaneous adipose tissue samples were snap-frozen in liquid nitrogen and stored at −80 °C for subsequent transcriptome and lipidomics analyses.

### 2.2. Analysis of Serum and Adipose Tissue

Serum samples were collected from all lambs in each group, and analyzed for hormone, enzyme, and metabolite concentrations and antioxidant capacities. Enzyme-linked immunosorbent assay (ELISA) kits (Shanghai BangYi Biological Technology Co., Ltd., Shanghai, China) were used to measure the hormones, adiponectin (BYE80146), leptin (BYE80165), insulin-like growth factor 1 (IGF1; BYE80149), growth hormone (GH; BYE93037), and cortisol (BYE93037), and the enzymes, fatty acid synthetase (FAS; BYE93016), acetyl-CoA carboxylase (ACC; BYE80134), lipase (BYE93219), stearyl-CoA desaturation enzyme (SCD; BYE80436), and lipoprotein lipase (LPL; BYE80455). They were measured according to the manufacturer’s instructions and using a microplate reader (Labsystems Multiskan MS 352, Thermo Fisher Scientific, Waltham, MA, USA). Commercial kits (Hunan Yonghe-Yangguang Science and Technology Co., Ltd., Hunan, China) were used to measure the serum metabolites, glucose (20192400113), triglyceride (TG; 20192400110), total cholesterol (TC; 20192400093), high-density lipoprotein cholesterol (HDL-C; 20192400088), low-density lipoprotein cholesterol (LDL-C; 20192400103), and creatinine (20192400117). They were determined following the manufacturer’s instructions and a biochemical analyzer (BS400, Mindray, Shenzhen, China). Serum FFAs were determined by colorimetry using a free-fatty-acid-assay kit (Shanghai BangYi Biological Technology Co., Ltd., Shanghai, China). Commercial kits (Shanghai BangYi Biological Technology Co., Ltd., Shanghai, China) were used to measure the antioxidant capacities of malondialdehyde (MDA; MS1401), glutathione peroxidase (GSH-Px; MS1202), superoxide dismutase (SOD; MS1500), total antioxidant capacity (TAC; MS1500), and a microplate reader.

### 2.3. Dorsal Subcutaneous Adipose Tissue Transcriptome Analysis

#### 2.3.1. RNA Isolation and Sequencing

Total RNA was isolated from dorsal subcutaneous adipose tissue samples of all lambs in each group according to the protocol recommended for the TRIzol reagent (Invitrogen, Waltham, MA, USA). The purity and quality of total RNA were determined by NanoPhotometer spectrophotometry (IMPLEN, München, Germany), and the integrity of the total RNA was determined using an Agilent 2100 bioanalyzer (Agilent Technologies, Santa Clara, CA, USA). Samples with total RNA integrity numbers (RIN) greater than 8 were used to construct cDNA libraries for sequence analysis. The sequencing libraries were generated using NEBNext^®^ UltraTM RNA Library Prep Kit for Illumina^®^ (NEB, Ipswich, MA, USA) following the manufacturer’s instructions, and index codes were added to attribute sequences to each sample. The clustering of the index-coded samples was performed on a cBot Cluster Generation System using TruSeq PE Cluster Kit v3-cBot-HS (Illumina, Ann Arbor, MI, USA) according to the manufacturer’s instructions. After cluster generation, the library preparations were sequenced on an Illumina Novaseq platform (Novogene Technology Co. Ltd., Beijing, China).

#### 2.3.2. Transcriptome Data Analysis

‘Clean’ sequence reads were produced by removing reads containing adapters, reads containing poly-N regions, and other low-quality reads from the raw read sequences. This was undertaken by calling accuracy, measured by the Phred quality score (Q score), with Q20 (a Q score of 20 is equivalent to the probability of an incorrect base call of 1%) and Q30 (probability of an incorrect base call of 0.1%), and the GC% content of the clean data were calculated.

All the subsequent analyses were based on the high-quality ‘cleaned’ data. The clean reads obtained were then mapped to an *Ovis aries* reference genome sequence [Oar_v4.0; https://www.ncbi.nlm.nih.gov/assembly/GCF_000298735.2/ (accessed on 9 October 2022)] using Hisat2 v2.0.5 (Center for Computational Biology, Johns Hopkins University, MD, USA). Fragments per kilobase of transcript per million mapped (FPKM) reads were calculated and normalized, based on the length of the gene, and reads count mapped to this gene. Differential-expression analysis of the four feeding groups (with four biological replicates per group) were performed using the DESeq2 R package (version 2_1.6.3;). Genes found at *p* < 0.05 using DESeq2 and |fold change| > 1 were assigned as differentially expressed genes (DEGs).

The Gene Ontology (GO) database [http://www.geneontology.org/ (accessed on 9 October 2022)] and the Kyoto Encyclopedia of Genes and Genomes (KEGG) [http://www.genome.jp/kegg/ (accessed on 9 October 2022)] were used to annotate the transcript sequences and analyze pathway enrichment of the DEGs using the cluster Profiler R package (version 4.0.0). The resulting *p*-values were adjusted using Benjamini and Hochberg’s approach for controlling the false discovery rate. A *P*_adj_ < 0.05 was used to identify GO and KEGG terms that were enriched by the up- and down-regulated DEGs.

### 2.4. Lipidomics Analysis

Total lipids were extracted from the subcutaneous adipose tissue from all the lambs in each group. Pre-cooled methanol (0.75 mL) was placed in a vortex-oscillating glass tube with a Teflon lined cap, 100 mg of lipid powder and 2.5 mL of pre-cooled methyl tertiary butyl ether (MTBE) were added, and the mixture was incubated at room temperature in a shaker for 1 h. Next, 0.625 mL of MS-grade water were added, and the contents were mixed and incubated at room temperature for 10 min to induce phase separation. The mixture was centrifuged at 1000 *g* for 10 min, the upper organic phase was collected, and a 1 mL solvent mixture (MTBE/methanol/water, 10:3:2.5, *v*/*v*/*v*) was added to the lower phase for re-extraction. The organic phase was collected again, pooled with the first collection, and then concentrated in a nitrogen-blowing desiccation apparatus (Reacti-Therm, Thermo Fisher Scientific, Waltham, MA, USA). Each extracted lipid sample was then dissolved in 100 μL isopropanol for LC-MS/MS analysis.

A VanquishTM UHPLC platform (Thermo Fisher Scientific, Waltham, MA, USA) was used to analyze the lipid samples. Aliquots of 5 μL were injected into a Thermo Accucore C30 column (Thermo Fisher Scientific) at a flow rate of 0.35 mL/minute using a 20-min mobile-phase-buffer-linear gradient with the column temperature set at 40 °C. The mobile-phase buffer ‘B’ included: acetonitrile/isopropanol (1/9) with 0.1% formic acid and 10 mM ammonium acetate. The chromatographic gradient elution procedure was as follows: initially, 30% B for 2 min, 30% B for 5 min, 43% B for 5.1 min, 55% B for 11 min, 70% B for 16 min, and 99% B for 18.1 min, followed by a 30% B run-through. A Q ExactiveTM HF mass spectrometer (Thermo Fisher Scientific) was operated in positive {negative} polarity mode with sheath gas: 20 arbitrary units, sweep gas: 1 arbitrary unit, auxiliary gas rate: 5 {7}, spray voltage: 3 kV, capillary temperature: 350 °C, heater temperature: 400 °C, S-Lens RF level: 50, resolving power (full scan): 120,000, scan range: 114–1700 *m*/*z*, automatic gain control target: 1e6, resolving power (MS2): 30,000 (Top20), normalized collision energy: 25; 30 {20; 24; 28}, injection time: 100 ms, isolation window: 1 *m*/*z*, automatic gain control target (MS2): 1e5, and dynamic exclusion: 15 s.

The raw data files were processed using Compound Discoverer 3.01 (CD3.1, Thermo Fisher) to determine peak alignment, peak picking, and quantitation for each metabolite. The main parameters were set as follows: retention-time tolerance, 0.2 min; actual mass tolerance, 5 ppm; signal-intensity tolerance, 30%; signal/noise ratio, 3; and minimum intensity, 100,000. Peak intensities were normalized to the total spectral intensity. The normalized data were used to predict the molecular formula based on additive ions, molecular ion peaks, and fragment ions, then the peaks were matched with the Lipidmaps and Lipidblast database to obtain accurate qualitative and relative quantitative readings. Pearson correlation coefficient analysis tested for relationships between quality control (QC) samples to ensure that the instrument was stable, and that the signal-response strength was normal when the experimental samples were detected. The quantitative data of the metabolites were converted logarithmically and standardized using the R package software, MetaX (version 4.0.0), and then the peaks extracted from all experimental samples were subjected to Univariate (UV) scaling for PCA analysis. When data were not distributed normally, transformations were done by the area normalization method. Candidate metabolites were filtered with variable importance of projection (VIP) > 1 and *p* < 0.01.

### 2.5. Correlation Analysis between Transcriptome DEGs and Lipid Metabolites

The top 100 DEGs with a |fold change| > 1 and *p* < 0.05, and the lipid metabolites with a VIP > 1 and *p* < 0.05 were used for correlation analysis in R (version 4.0.0). The Spearman correlation coefficient, *p* value, and *P*_adj_ of each DEG relative FPKM, and each lipid-relative-quantitative value in each sample were calculated by the “rcorr” package in R (version 4.0.0), and drawings were created with the R package.

### 2.6. Aqueous Extraction of Agriophyllum SQUARROSUM and Lamb Adipocyte Culture

The aqueous extraction of *A. squarrosum* followed the method described by Bao et al. [16]. Lamb-inguinal-adipose tissue was cut into 1 mm^3^ pieces using sterile scissors, placed into a centrifuge tube, and mixed with an equal volume of 0.1% type I collagenase (Sigma, St. Louis, MO, USA). The mixture was oscillated and digested at 37 °C for 1 h, and then was added to an equal volume of complete-culture medium to terminate digestion, as described by Li [17]. The digestive fluid was filtered through successive 300 and 100 mesh screens, centrifuged at 720 *g* for 8 min, and then the supernatant was discarded. The sediment was washed with the complete-culture medium twice, and re-suspended. A total of one mL of aliquots of the re-suspended cell solutions with an equal volume of complete-culture medium were added to a 96-well plate (191-6381, Crystalgen Incorporated, Commack, NY, USA). The cell culture was kept in an incubator (XD-101, Sanyo, Osaka, Japan) at 37 °C and 5% CO_2_, with the culture medium replaced every 2 days until cell coverage reached 80–90%.

Different concentrations of the aqueous extract of *A. squarrosum* were used to treat the cultured adipocytes, with the concentrations based on previous studies where flavonoids from plants were used on 3T3-L1 mice adipocytes [18,19]. In the present study, 0, 50, 100, and 200 μg/mL of *A. squarrosum* aqueous extracts were added to the complete culture medium for treating the lamb adipocytes.

When the primary culture of lamb adipocytes grew to approximately 90% confluence in the well plates, the cells were digested with 0.25% trypsin-ethylene diamine tetra-acetic acid (EDTA) (16400-044, Gibco, Amarillo, TX, USA), and the cell concentration was adjusted to 5 × 10^4^/mL. An aliquot of 100 μL of the cells were then seeded into a new 96-well plate with 4 replicates allocated per treatment (complete culture medium containing different concentrations of *A. squarrosum* extract) as described above, and cultured for 24 h. The culture medium containing the different concentrations of *A. squarrosum* extract was then replaced and the cells were cultured for another 48 h. Next, the 100 μL of culture medium were replaced with a mixture containing 10 μL cholecystokinin-8 (CCK-8) (C0037, Beyotime Biotechnology, Haimen, China), cultivated for 15–30 min, and then absorbance was measured at 450 nm with a microplate reader (MK3, Thermo Fisher Scientific) to determine the amount of cell proliferation.

After 72 h, 90 μL of fresh-basal-culture medium and 10 μL of methyl thiazolyl tetrazolium (MTT) (Sigma, St. Louis, MO, USA) were added to each well, and incubated at 37 °C and 5% CO_2_ for 3 h in the dark. The liquid in the well was aspirated off and 200 μL of dimethylsulfoxide (DMSO) (Invitrogen) were added and shaken at room temperature for 10 min. Absorbance was measured at 570 nm with a microplate reader (MK3, Thermo Fisher Scientific) to determine cell proliferation. Four replicate wells with cell-free reaction mixture served as the negative control in the measurement of CCK8 and MTT, separately.

When the primary cultured-lamb adipocytes grew to approximately 100% confluence in the well plates, they could be differentiated into adipocytes as described by Li [17]. To do this, the cells were cultured for 48 h and then transferred to differentiation medium 1 (90% DMEM; 10% FBS; IBMX: 0.5 mM; Dex: 1 μM; insulin: 10 μg/mL) and cultured for another 48 h. They were then transferred to differentiation medium 2 (insulin: 10 μg/mL; high-glucose DMEM [10% FBS]) and cultured for another 48 h. The culture medium containing 10% FBS and high-sugar DMEM was replaced once a day and the cells cultured for 4 d in total. The culture medium was then aspirated, the cells were washed twice with PBS, fixed for 2 min, and stained with oil-red O to distinguish cell differentiation.

#### 2.6.1. Lamb Adipocyte Metabolism in *Agriophyllum squarrosum* Extract Culture

Lamb adipocytes in the different treatment groups were assayed for glycerol phosphate dehydrogenase (GPDH) content, triglyceride content, and FFA content using the GPDH Activity Assay Kit (MAK208, Sigma-Aldrich, St. Louis, MO, USA), Triglyceride Quantification Colorimetric/Fluorometric Kit (MAK266, Sigma-Aldrich, St. Louis, MO, USA), and Free Fatty Acid Quantitation Kit (MAK044, Sigma-Aldrich, St. Louis, MO, USA), following the manufacturer’s protocols.

#### 2.6.2. Real-Time PCR of Lamb Adipocytes in *Agriophyllum squarrosum* Extract Culture

Total RNA was isolated from the cultured-lamb adipocytes with TRIzol reagent (Invitrogen, ThermoFisher Scientific, Waltham, MA, USA), and 1 μg of total RNA was subjected to first-strand cDNA synthesis with oligo-dT (deoxythymidine) primers and Superscript II reverse transcriptase (Invitrogen). The primer was designed using Primer 6 (Palo Alto, CA, USA); the primer sequences of the genes are presented in Table 2. The mRNA levels were normalized against expression of the housekeeping gene GAPDH, and the relative mRNA levels were calculated employing the 2^−△△ct^ method.

### 2.7. Statistical Analyses

The Levene’s test tested the data for normal distribution of variance. Date distributed normally were analyzed by one-way analysis of variance (ANOVA; IBM SPSS Statistics 20, Chicago, IL, USA), while data with non-normal distribution were analyzed by the non-parametric Kruskal-Wallis test. Duncan’s test separated means when significance was detected. Polynomial contrasts tested whether the effect of *A. squarrosum* on the variable measured was linear, quadratic, or cubic with an increase in intake, and *p* < 0.05 was accepted as the level of significance.

## 3. Results

### 3.1. Agriophyllum squarrosum Affects Blood Metabolism and Adipose Tissue Morphology

**Figure 1:**
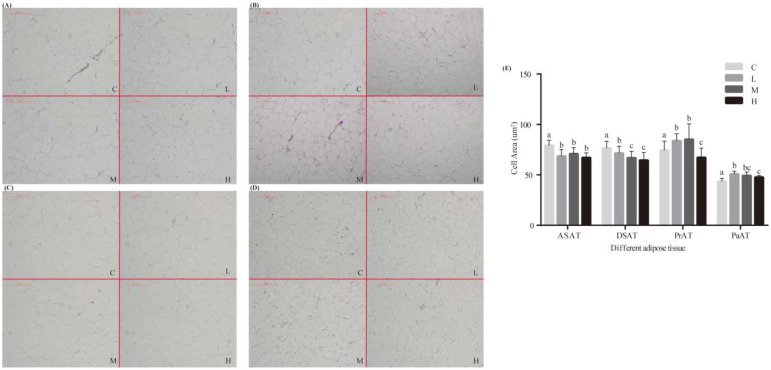
Effects of *Agriophyllum squarrosum* on different adipose tissue histomorphologies and cell areas in Tan lambs. C, control; L, 10% *A. squarrosum* diet; M, 20% *A. squarrosum* diet; H, 30% *A. squarrosum* diet. (**A**–**D**): effects of *A. squarrosum* on adipose-tissue morphology. (**A**), ASAT: abdominal subcutaneous adipose tissue. (**B**), DSAT: dorsal subcutaneous adipose tissue. (**C**), PrAT: peri-renal adipose tissue. (**D**), PaAT: peri-aortic adipose tissue. (**E**): effects of *A. squarrosum* on adipose-tissue cell areas. a, b, c Means with different letters differ from each other (*p* < 0.05).

Supplementary *A. squarrosum* did not affect feed intake, average daily gain, and body weight [14], but caused changes in serum metabolites (Table 3). The serum concentrations of total cholesterol (TC) were lower (*p* = 0.004) in the 30% supplemented lambs than the controls, while concentrations of high-density lipoprotein cholesterol (HDL-C) were lower (*p* = 0.006) in the 10% and 20%, but not in the 30% supplemented lambs, than controls. Cortisol concentrations were lower (*p* = 0.012) in the 30% supplemented lambs, and FFA concentrations were higher in the 10% and 20% supplemented lambs than controlled lambs (*p* < 0.001). Significant quadratic effects were detected for serum concentrations of MDA (*p* < 0.001) and LDL-C (*p* < 0.001), with MDA peaking at 10% supplementation and then decreasing, whereas LDL-C decreased till 20% supplementation and then increased. The area of adipocytes in subcutaneous-adipose tissue (abdominal-subcutaneous-adipose tissue and dorsal-subcutaneous-adipose tissue) decreased (*p* < 0.05) with supplementary *A. squarrosum*, but there was no difference between the 20% and 30% supplements. However, the area in visceral-adipose tissue (peri-aortic adipose tissue and peri-renal adipose tissue) increased (*p* < 0.05), especially with 10% and 20% supplements (Figure 1).

### 3.2. Effects of Agriophyllum squarrosum on Immune and Metabolism-Related Gene Expressions in Adipose Tissue

**Figure 2:**
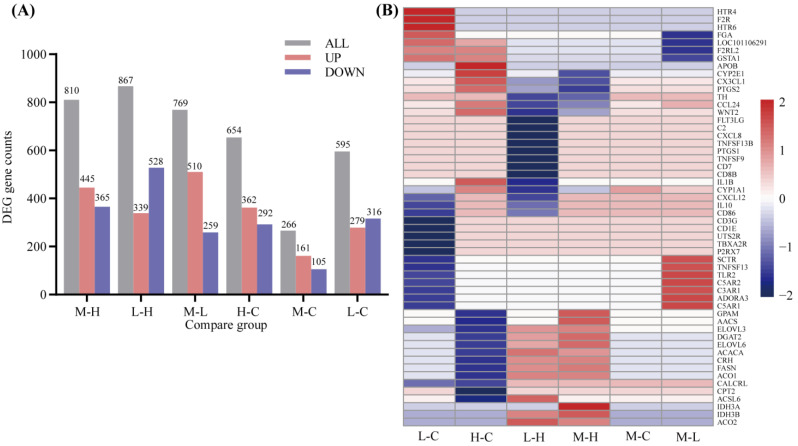
Transcriptome differentially expressed genes (DEGs) expression and clustering in Tan lambs. (**A**) Histogram of DEGs expression among different comparison groups. Pink represents up-regulated DEGs, blue-violet represents down-regulated DEGs. (**B**) Heatmap generated from DEGs and candidate genes using the hierarchical clustering (Pearson-distance-clustering algorithm) features in the R. C, control; L, 10% *Agriophyllum squarrosum* diet; M, 20% *A. squarrosum* diet; H, 30% *A. squarrosum* diet.

The sequencing quality results are presented in Table S1. The transcriptome analysis suggested that the 20% diet was closer to the control group than the other levels (Figure S1A). In total, there were 2470 DEGs. The 10% diet regulated 595 DEGs compared to the control diet, and the 20% diet regulated 769 DEGs and 810 DEGs compared to the 10% and 30% diets, respectively (Figure 2A). Hierarchical-clustering analysis of all the annotated genes indicated that mRNA expression differed among groups (Figure S2), and the heatmap demonstrated that there was a significant difference between the 20% diet and the other diets in DEGs (Figure 2B).

**Figure 3:**
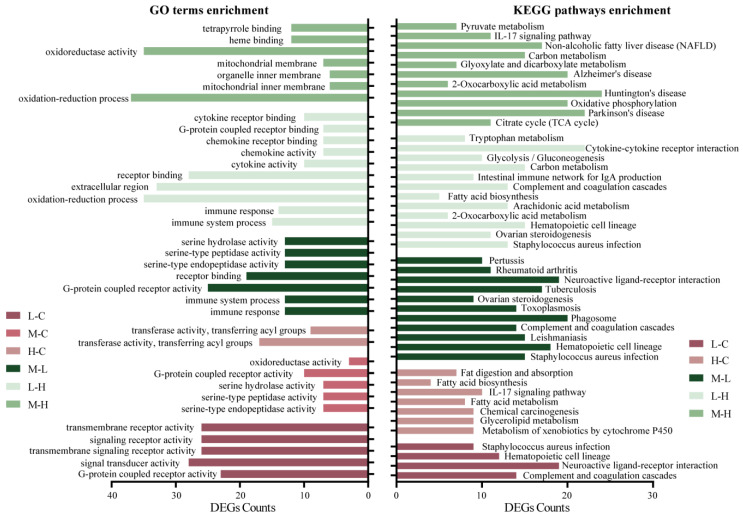
The significantly enriched Gene Ontology (GO) terms and Kyoto Encyclopedia of Genes and Genomes (KEGG) pathways for differentially expressed genes (DEGs) in the different groups of *Agriophyllum squarrosum* supplemented Tan lambs. C, control; L, 10% *A. squarrosum* diet; M, 20% *A. squarrosum* diet; H, 30% *A. squarrosum* diet.

In the present study, supplementary *A. squarrosum* altered the expression of genes involved in cell differentiation and proliferation, immunity and metabolism in the adipose tissue of lambs, such as hematopoietic-cell lineage, complement, and coagulation cascades, and lipid metabolism. With the KEGG pathways and GO terms analysis, the DEGs were enriched significantly in immune and inflammatory factors (i.e., 20% vs. 10%, 10% vs. 0% and 10% vs. 30%: Table S2) and metabolism process (i.e., 30% vs. 0% and 20% vs. 30%: Table S2) in the groups that were compared (Figure 3). Compared to the control diet, there was no significant enrichment in KEGG pathways in the 20% diet.

### 3.3. Effects of Agriophyllum squarrosum on Lipid Metabolism in Tan Lambs

Differences in lipid profiles were analyzed using lipidomics analyses, which showed that the quality of replicates within groups was appropriate for subsequent analyses (Figure S1B). Partial least squares discrimination analysis (PLS-DA) models were applied to validate the fitness and predictability of the metabolomics models. Validation parameters of fitness (R2X = 1 and R2Y > 0.85) and R2 > Q2 were used for comparison between groups (Figure S1C).

**Figure 4:**
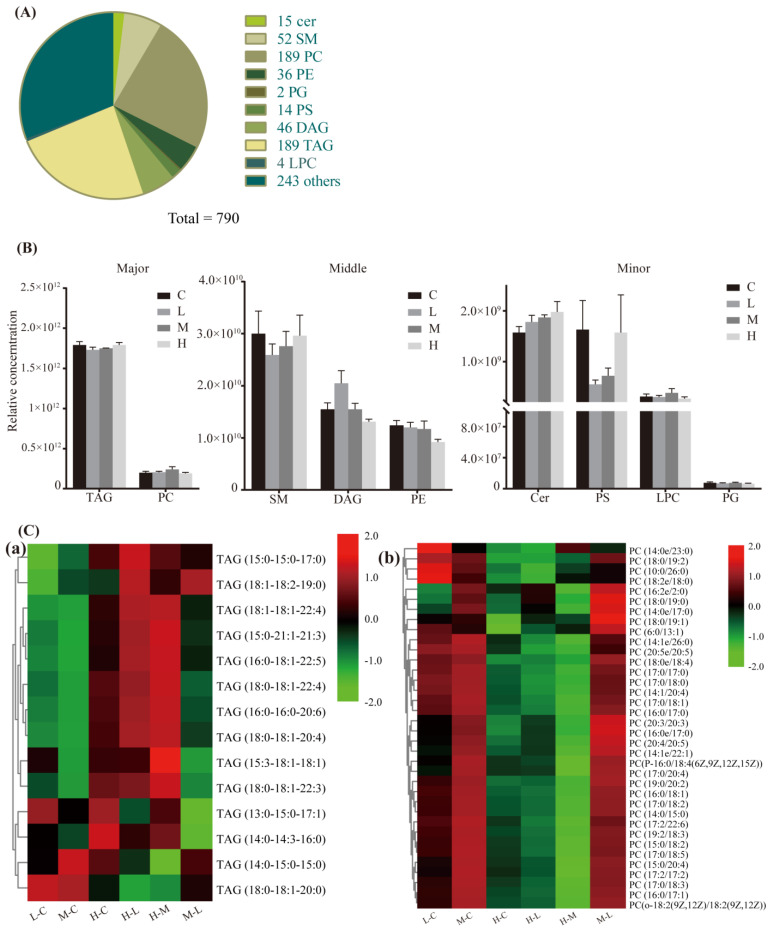
General analysis of dorsal subcutaneous adipose tissue (DSAT) lipidomics profiles in Tan lambs. (**A**) Overall distribution of major lipid classes DSAT, n ≥ 5 per group. (**B**) Total relative content of every lipid class. (**C**) Heatmap generated from two major lipids of triacylglycerol (TAGs) (a) and phosphatidylcholines (PCs) (b) using hierarchical clustering (Pearson-distance-clustering algorithm) in TBtools. C, control; L, 10% *Agriophyllum squarrosum* diet; M, 20% *A. squarrosum* diet; H, 30% *A. squarrosum* diet.

A total of 790 lipid metabolites from the 10 lipid classes were identified in the dorsal subcutaneous adipose tissue of 0%, 10%, 20%, and 30% supplemented diets, including ceramides (Cer), sphingomyelins (SM), phosphatidylcholines (PC), phosphatidylethanolamines (PE), phosphatidylglycerol (PG), phosphatidylserines (PS), lyso-PC (LPC), triacylglycerol (TAG), diacylglycerol (DAG), and others (Figure 4A). A total of 103 differential lipids were screened out from the 10 lipid classes. The total relative content of the 10 lipid classes did not differ among diets (Figure 4B). However, as illustrated in the heatmap (Figure 4C), there were differences in lipids among all diets. Three differential triacylglycerols decreased and three increased, and four differential ceramides increased in the supplemented diets compared to the control group, while 10 differential triacylglycerols, 34 differential phosphatidylcholines, and seven differential phosphatidylethanolamines decreased in the 30% diet compared to the other diets.

### 3.4. Agriophyllum squarrosum Regulates Lipid Profiles and DEGs Involved in Immune and Lipid Metabolism in Adipose Tissue of Tan Sheep

The results of the combined analysis of the top 100 DEGs and significantly different lipidomic profiles are presented in Figure S3. The PSs decreased significantly only in the 10% diet compared to the control diet, and was correlated negatively with the up-regulated DEG NUAK family kinase 2 (*NUAK2*) (r > 0.85, *P_adj_* < 0.05), and pleckstrin homology, such asdomain family A member 1 (*PHLDA1*) and glutathione S-transferase alpha 1 (*GSTA1*) (r > 0.75, *p* < 0.05). The significantly increased Emblicanin B was correlated negatively with the down-regulated sclerostin (*SOST*) gene (r > 0.85, *P_adj_* < 0.05) in the 10% diet compared to the control diet. The 11-nitro-1-undecene in the 30% diet decreased significantly compared to the control diet, and was correlated positively with the down-regulation of acetoacetyl-CoA synthetase (*AACS*) (r > 0.85, *P_adj_* < 0.05). The oleanolic acid in the 30% diet increased significantly compared to the control diet, and was correlated negatively with the down-regulation of angiopoietin, such as 8 (*ANGPTL8*) and NADH dehydrogenase (ubiquinone) complex I (*NDUFAF1*) (r > 0.85, *P_adj_* < 0.05).

### 3.5. An Agriophyllum squarrosum aqueous Extract Promotes Adipocyte Proliferation, Inhibits Adipocyte Differentiation, and Is Involved in Inflammatory Response in Tan Lambs

**Figure 5:**
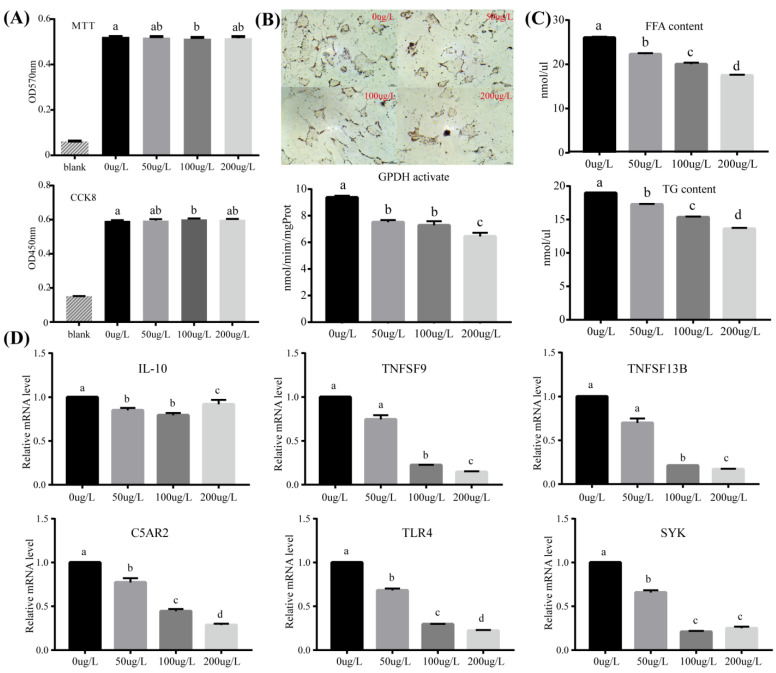
Effects of *Agriophyllum squarrosum* extract on lamb-adipocyte tissue: (**A**) proliferation; (**B**) differentiation; (**C**) lipid metabolism; and (**D**) immune and inflammatory responses for selected genes. Means with different letters differ from each other *p* < 0.05.

The results indicated that as little as 100 μg/L of *A. squarrosum* aqueous extract promoted adipocyte proliferation (Figure 5A), and all tested levels of *A. squarrosum* extract inhibited adipocyte differentiation and decreased the expression of GPDH during adipocyte differentiation (Figure 5B). Different concentrations of *A. squarrosum* extract significantly reduced lamb-adipocyte triglycerides and free-fatty-acids contents (Figure 5C). Immune and inflammatory response-related genes interleukin (*IL10*), complement anaphylatoxin C5a receptor 2 (*C5AR2*), toll-like receptor 4 (*TLR4*), spleen tyrosine kinase (*SYK*), tumor necrosis factor superfamily member 9 (*TNFSF9*), and *TNFSF13B* also decreased expressions with *A. squarrosum* aqueous extract (Figure 5D).

## 4. Discussion

As a potential food crop, seeds of *Agriophyllum squarrosum* have comparable nutritive value to quinoa, its relative species, and it has been consumed by the Chinese for over a 1000 years [20]. The seeds and above-ground parts have been used in folk medicine for detoxification, as antipyretic and analgesic agents, and to cure diuresis and combat plagues [1]. Although the above-ground biomass of *A. squarrosum* is small per plant, it has a relatively high community biomass of 26.1 g dry matter per m^2^ [21], which could potentially provide high-quality forage for livestock. There is, however, no report of its use as feeds for sheep. In the present study, we applied RNA-seq, mass-spectrometry-based lipidomics analyses, and adipocyte metabolism to describe the molecular signature at the levels of the transcriptome, lipidome, and cell for the first results of the effect of *A. squarrosum* on sheep metabolism.

### 4.1. Agriophyllum squarrosum Acts as a Hypolipidemic Agent and Regulates Lipid Metabolism

In the present study, *A. squarrosum* decreased serum concentrations of total cholesterol, high-density lipoprotein cholesterol, low-density lipoprotein cholesterol, and cortisol, especially when supplemented at 30% of the diet. In the transcriptome analysis, the corticotropin releasing hormone (*CRH*) and corticotropin releasing hormone receptor 2 (*CRHR2*) gene were down-regulated in the 30% diet compared to the control diet, and immuno-inflammatory responses were decreased in the 10% and 30% diets, which could cause a decrease in serum cortisol. Zhaorigetu et al. [22] also reported that extracts of *A. squarrosum* lowered serum-lipid concentration in rats, specifically, concentrations of total cholesterol, triglycerides, and low-density lipoprotein cholesterol. However, in the present study, the serum concentration of free-fatty acids increased with higher levels of *A. squarrosum* supplementation, but decreased in lamb adipocytes. This is presumably because the addition of *A. squarrosum* inhibited the synthesis of adipose tissue, resulting in an increased serum concentration of free fatty acids.

Supplementary *A. squarrosum* decreased the size of sheep subcutaneous adipocytes and increased the size of visceral adipocytes. Large adipocytes are normally hyper-lipolytic and resistant to the anti-lipolytic effect of insulin [23]. Visceral adipocyte tissue contains a greater number of large adipocytes than subcutaneous adipocyte tissue, which contains small adipocytes. Small adipocytes are reported to be insulin-sensitive with a high affinity for the uptake of free fatty acids and triglycerides, preventing their deposition in non-adipose tissue [24,25]. This suggests that *A. squarrosum* may affect the allocation of energy to different tissues in sheep. Although it was demonstrated that *A. squarrosum* could reduce elevated blood-glucose concentration in diabetic mice [16], there was no apparent effect of *A. squarrosum* on serum glucose concentration in the present study, which likely reflects that the lambs were healthy and non-diabetic.

The seeds of *A. squarrosum* are rich in antioxidant constituents [3], and these may play a role in improving liver and kidney functions [6]. It was reported that antioxidants were released upon cooking in an in vitro digestion experiment [26], but in the present study, a serum concentration change in the antioxidant indicators GP-x, SOD and TAC with *A. squarrosum* supplementation was not detected. However, it is difficult to draw conclusions in comparisons between humans and ruminants, as only *A. squarrosum* seeds are consumed by humans and the whole plant is consumed by ruminants. A change in serum MDA concentration, an end product of lipid peroxidation, is considered to be a reliable indicator of oxidative stress [27]. Lipid peroxidation is a cause of cell-membrane disruption [28], cell damage [29], metabolic disorders, and inflammation [30,31]. In the present study, the serum MDA concentration increased with the 10% supplementation diet, but the oxidative stress-related genes, prostaglandin endoperoxide synthase 1 (*PTGS1*) and prostaglandin endoperoxide synthase 2 (*PTGS2*), decreased in the 10% compared to the 30% diet. Previous studies found that prostaglandin endoperoxide synthase (PTGS), the key enzyme in inflammatory reaction, regulates inflammation caused by oxidative stress, and is correlated positively with adipose tissue inflammation and MDA concentration [32,33]. The different results between the present study and previous studies and the metabolic relationship between blood and adipose tissue warrants further study.

### 4.2. Transcriptomics Analysis Reveals That Agriophyllum squarrosum Regulates Candidate Genes Involved in Immune and Lipid Metabolism

Previous studies in animals fed *A. squarrosum* focused on bodyweight changes and serum metabolite concentrations (14). In the current study, we examined the transcriptional regulation of *A. squarrosum* on sheep adipose tissue. We found that the 10% supplementation diet expressed more DEGs in the immune system, including complement receptor type 2 (*CR2*), complement factor D (*CFD*), complement component 3a receptor 1 (*C3AR1*), complement component 5a receptor 1 (*C5AR1*), tumor necrosis factor superfamily member 13 (*TNFSF13*), toll-like receptor 2 (*TLR2*), and *CD 86*. The transcripts for the *CR2*, *C3AR1*, *CFD*, *C5AR1*, *TNFSF13*, and *TLR2* were down-regulated in the 10% group compared to the control diet (Figure 3C). It was reported that up-regulation of *C3AR1* [34], *CFD* [35], and *C5AR1* [36] all promoted inflammatory responses, and *TLR2* was associated with downstream NF-κB activation and pro-inflammatory cytokine production in adipocytes [37]. The activation of Toll-like receptors leads to up-regulation of several important cell-surface molecules (*CD86* and *TNFSF13*) that promote inflammatory responses.

The 30% diet regulated more DEGs involved in metabolism, especially in lipid metabolism, including candidate genes diacylglycerol O-acyltransferase 2 (*DGAT2*), glycerol-3-phosphate acyltransferase (*GPAM*), ELOVL fatty acid elongase 3 (*ELOVL3*), ELOVL fatty acid elongase 6 (*ELOVL6*), apolipoprotein B (*APOB*), fatty acid synthase (*FASN*), acetoacetyl-CoA synthetase (*ACACA*), aconitase 1 (*ACO1*), aconitase 2 (*ACO2*), and acyl-CoA synthetase short-chain family member 2 (*ACSS2*). The expression of *DGAT2*, *ELOVL3*, *ELOVL6*, and *FASN* were down-regulated in the 30% group compared to other groups, and *CPT2* and *GPAM* were down-regulated significantly in the 30% group compared to the control diet. *DGAT2*, *ELOVL3*, *ELOVL6*, *FASN, GPAM, ACACA*, and *ACCS2* genes were involved in lipid synthesis [38,39,40,41,42,43]. *ACO1* is the rate-limiting enzyme for β-oxidation of long-chain saturated fatty acids peroxidase, whereas *ACO2* oxidizes branched-chain fatty acids [44], and *APOB* is reputed to be associated with lipid disorders and inflammatory responses [45]. The present study demonstrated that the 30% diet regulated lipid metabolism and was involved in the immune system. *CFD* and *C3AR1* genes were down-regulated in the 30% group compared to the 10% diet (Figure 3C), which suggested that these genes also play key roles in lipid metabolism in the 30% diet, as *CFD* promotes adipocyte differentiation and lipid synthesis by inducing C3A-receptor-target gene expression [46]. The down-regulation of these DEGs and the enrichment of lipid metabolism pathways in the 30% diet compared to the other groups implied that the 30% diet inhibited lipid synthesis, which supported the smaller subcutaneous adipocytes and increased serum-free-fatty-acids concentration in the present study. The inhibition of oxidative utilization might be related to the reduction of lipid metabolism and inflammatory responses. The 30% diet also down-regulated energy metabolism related DEGs, including fructose-bisphosphatase 2 (*FBP2*) and isocitrate dehydrogenase 3 (NAD+) beta (*IDH3B*) compared to the 20% and 10% diets, which were involved in gluconeogenesis [47] and tricarboxylic acid cycle [48].

### 4.3. Agriophyllum squarrosum Involved in Immune Regulatory Response and Lipid Metabolism in Adipocytes

We further confirmed the metabolic regulation of *A. squarrosum* on the adipose tissue by the growth of adipocytes and gene expression of *A. squarrosum* aqueous extracts. With the addition of *A. squarrosum* to adipocytes, the inflammation-related genes, *C5AR2*, *TLR4*, *SYK*, *TNFSF9*, and *TNFSF13B*, were down-regulated, and there was an increase in the down-regulation of immune-related genes with an increased content in the extract added. These results suggested that *A. squarrosum* had a strong immune regulatory effect. Previous studies reported that the inflammatory state of adipose tissues has substantial effects on lipid metabolism, including decreasing triglycerides synthesis and increasing lipolysis [49]. These processes, in turn, result in decreased concentrations in adipocytes and increased concentrations of circulating-free-fatty acids, as observed in the present study.

### 4.4. Agriophyllum squarrosum Regulates Lipid Profiles Involved in Immune and Anti-Inflammatory Responses

Using mass spectrometry, we determined that *A. squarrosum* regulated triacylglycerols, diacylglycerols, phosphatidylcholines, phosphatidylserines, and bioactive-small molecules involved in enhancing immunity, anti-inflammatory responses, and lipid metabolism. Although there was no difference in the total relative content of the 10 lipid classes, the lipid substances differed between comparison groups. The greater concentration of short-chain triglycerides in the supplemented *A. squarrosum* groups than the control group suggests that a low dose of *A. squarrosum* altered the carbon chain length of lipids. The increased serum concentration of triglycerides in the 30% diet compared to the other diets might be related to the decreased expression of long-chain lipid syntheses DEGs, according to the transcriptome analysis. The decreased concentration of diacylglycerols in the 10% and 30% diets, and the increased immune response in the 20% diet may have been caused by the increased phosphatidylcholine content. The large accumulation of intermediates of lipid metabolism, such as diacylglycerols and phosphatidylcholines, are considered to be detrimental to health and they induce inflammatory responses [50]. Metabolic flux through the de novo phosphatidylcholine synthesis pathway and cellular phosphatidylcholine concentrations are increased greatly in differentiating macrophages [51]. Furthermore, pro-inflammatory signaling via *TLR4* increases the rate of choline uptake and de novo phosphatidylcholine synthesis in macrophages [52,53].

Inflammatory responses induced by apoptotic cells were mitigated by the inhibition of phosphatidylserine and vitronectin receptors [54], which suggested that phosphatidylserines were associated closely with apoptosis and inflammatory responses. Furthermore, phosphatidylserines play a role in adipocyte-macrophage homeostasis, an important part of which is the regulation of inflammation in white adipose tissue recruits and activation of macrophages to promote the secretion of inflammatory adipokines, when phosphatidylserine is exposed to adipose cellularity under hypoxic stress [55]. In the present study, the relationships between phosphatidylserines, apoptosis, and lipid metabolism-related genes, namely, *NUAK2* [56] and *GSTA1* [57], and *PHLDA1* [58,59], suggested that decreased concentrations of phosphatidylserines may protect against apoptosis-induced inflammation and obesity in the adipose tissue.

### 4.5. Lipid Metabolites of Agriophyllum squarrosum Involved in Important Health Function

Several lipids were identified to have medicinal properties. The positive correlation between 11-nitro-1-undecene and the *AACS* gene [60] suggests that 11-nitro-1-undecene is linked to lipid deposition. Inflammation and related gene expression in WAT correlated positively with adipocyte size and body mass index (BMI) in humans, and bigger adipocytes indicated deposition of more lipids. The reduction of lipid deposition could alleviate stimulation of adipose tissue to inflammatory responses [61]. The 11-nitro-1-undecene was also detected in the traditional medicinal plant, *Cynanchum paniculatum*, which has similar effects on lipid metabolism as *A. squarrosum* [62]. Interestingly, active components of small molecule lipids were identified in the current study. Oleanolic acid, which increased in the 30% diet, has derivatives that possess promising pharmacological uses, such as hepato-protective, [63], antioxidant [64], anti-inflammatory and anti-cancer properties [65,66]. In the present study, the negative correlations between oleanolic acid and the *ANGPTL8* [67] and *NDUFAF1* [68] genes were related to the decreased lipid storge and increased glycolytic activity in adipose tissue. In addition, emblicanin B, a low molecular weight hydrolyzable tannin (< 1000), along with pedunculagin and punigluconin, was the key ingredients in emblica-cascading antioxidant [69]. It correlated negatively with *SOST* [70], which indicated that oleanolic acid related to the inhibition of lipid deposition. In summary, *Agriophyllum squarrosum* not only regulates lipid metabolism and immune responses, but is also involved in important health functions.

## 5. Conclusions

*Agriophyllum squarrosum* reduced blood lipids, enhanced immune responses, and regulated the metabolism of lipids and glucose in adipose tissue of Tan lambs. The present study provides strong evidence that *A. squarrosum* has the potential to be used as a beneficial dietary supplement for ruminants. A diet containing 10% *A. squarrosum*, on a DM basis, is recommended, but further research is required to determine the precise optimal level.

## Figures and Tables

**Table 1:** Composition of the pellets offered the four groups of Tan lambs [14].

Ingredient (g/100 g)	Diet (% *Agriophyllum squarrosum*)			
	0%	10%	20%	30%
Corn straw	16.05	10.7	5.35	0
Alfalfa hay	13.95	9.3	4.65	0
*Agriophyllum squarrosum*	0	10	20	30
Corn kernels	40.1	40.1	40.1	40.1
Wheat bran	5.6	5.6	5.6	5.6
Soybean meal	10.5	10.5	10.5	10.5
Cotton-seed meal	6.9	6.9	6.9	6.9
Molasses	2.8	2.8	2.8	2.8
Limestone flour	1	1	1	1
Sodium chloride	0.7	0.7	0.7	0.7
Sodium bicarbonate	1.4	1.4	1.4	1.4
Premix compound ^1^	1	1	1	1
**Nutritional level**				
Metabolizable energy (MJ/kg)	11.21	11.54	10.69	8.87
Dry matter (% DM)	88.84	89.28	88.58	89.38
Crude protein (% DM)	18.20	18.57	18.62	18.18
Neutral-detergent fiber (% DM)	31.61	34.53	39.14	39.33
Acid-detergent fiber (% DM)	15.95	18.60	23.52	25.88
Carbohydrates (% DM)	40.24	36.23	31.33	31.28
Crude fat (% DM)	1.61	1.71	1.68	1.58
Ash (% DM)	8.34	8.96	9.23	9.63

^1^ Each kg of premix contains 400,000 IU vitamin A, 40,000 IU vitamin D, 8000 IU vitamin E, 3600 mg iron, 4000 mg zinc, 400 mg copper, 2000 mg manganese, 20 mg selenium, 20 mg cobalt and 40 mg iodine. DM = dry matter.

**Table 2:** Target genes and the primer sequences used in RT-PCR analysis of lamb adipocytes.

Gene Name	Primer Sequences	Tm (°C)
*TNFSF9-F*	5′-CTTGACTCTGAAGCGCGTGA-3′	60
*TNFSF9-R*	5′- AGAGCTGCCAGGAGAGA-3′	
*TNFSF13B-F*	5′-GAGGCCGAAGAAACAGTC-3′	60
*TNFSF13B-R*	5′-TCCCATGGCAAAGGTGTTA-3′	
*IL10-F*	5′-AGGACCAACTGAACAGCATG -3′	60
*IL10-R*	5′-GAGTTCACGTGCTCCTTGAT-3′	
*SYK -F*	5′-TCCGTCCACAACTTCCAAGT-3′	60
*SYK-R*	5′-CCTAGTTCCTTGTCTTCCAG-3′	
*TLR4 -F*	5′-CGGCATTTCACTCCCTCCCT-3′	60
*TLR4-R*	5′-CTTGACCCACTGCAGGAAAC-3′	
*C5AR2-F*	5′-CTGTCATCCTGCTCTCCATG-3′	60
*C5AR2-R*	5′- ACCGTAGTCCACCACACACT-3′	
*GAPDH -F*	5′-CTGCCCGTTCGACAGATAGC-3′	60

**Table 3:** Effects of 0% (Control), 10%, 20% and 30% *Agriophyllum squarrosum* on serum-biochemical indices in Tan lambs (means ± SEM).

	0% (Con)	10%	20%	30%	SEM	*p* Value			
						Treatment	Linear	Quadratic	Cubic
MDA (mmol/mL)	8.80 ^a^	11.01 ^b^	9.45 ^a^	8.63 ^a^	0.26	0.001	0.102	0.001	0.663
GSH-Px (U/mL)	840	880	881	877	34.6	0.977	0.759	0.770	0.063
SOD (U/mL)	47.8	62.6	58.2	58.7	3.47	0.537	0.434	0.330	0.066
TAC (U/mL)	15.2	14.8	15.1	13.9	0.45	0.788	0.444	0.684	0.454
TG (mmol/L)	1.60	1.48	1.67	1.62	0.03	0.208	0.358	0.643	0.486
TC (mmol/L)	5.60 ^a^	5.08 ^ab^	5.33 ^a^	4.41 ^b^	0.14	0.010	0.004	0.366	0.766
HDL-C (mmol/L)	1.69 ^a^	1.48 ^b^	1.45 ^b^	1.45 ^b^	0.03	0.011	0.006	0.047	0.006
LDL-C (mmol/L)	3.31 ^a^	3.02 ^b^	2.96 ^b^	3.38 ^a^	0.05	0.002	0.497	<0.001	0.002
FFA (mmol/L)	0.66 ^a^	0.73 ^a^	1.12 ^b^	0.92 ^b^	0.05	0.001	<0.001	0.024	0.913
Adiponectin (μg/L)	3388	3372	3425	3380	88.15	0.997	0.974	0.936	0.446
ACC (U/mL)	374	394	363	385	8.26	0.580	0.990	0.946	0.605
FAS (U/mL)	2321	2296	2124	2330	67.61	0.693	0.844	0.412	0.846
GH (ng/mL)	8.51	8.81	8.70	8.68	0.25	0.984	0.892	0.770	0.172
IGF-1 (ng/mL)	180	172	187	182	6.37	0.876	0.726	0.917	0.970
Leptin (ng/mL)	8.29	8.89	8.89	8.39	0.27	0.818	0.960	0.348	0.501
Lipase (U/mL)	129	129	137	128	3.77	0.848	0.858	0.589	0.469
LPL (U/mL)	117	113	112	117	3.20	0.911	0.993	0.482	0.701
SCD (U/mL)	66.6	68.2	71.7	68.7	2.32	0.908	0.680	0.648	0.834
Glucose (mmol/L)	4.24	4.85	4.87	4.76	0.12	0.257	0.186	0.144	0.903
Creatinine (μmol/L)	106	100	97	104	2.21	0.540	0.733	0.172	0.405
Cortisol (ng/mL)	298 ^a^	276 ^ab^	282 ^a^	239 ^b^	7.84	0.042	0.012	0.424	0.216

^a, b,^ Means in a row with different letters differ from each other at *p* < 0.05. MDA: malondialdehyde, GSH-Px: glutathione peroxidase, SOD: superoxide dismutase, TAC: total antioxidant capacity, TG: triglyceride, TC: total cholesterol, HDL-C: high-density lipoprotein cholesterol, LDL-C: low-density lipoprotein cholesterol, FFA: free fatty acids, ACC: acetyl-CoA carboxylase, FAS: fatty acid synthetase, GH: growth hormone, IGF-1: insulin-like growth factor 1, LPL: lipoprotein lipase, SCD: stearyl-CoA desaturation enzyme.

## Data Availability

The data are available confidentially to editors and reviewers, and all transcriptome data were submitted to the NCBI Sequence Read Archive (SRA series accession: RJNA753589).

## Supplementary Materials

The following supporting information can be downloaded at: https://www.mdpi.com/article/10.3390/ani12243486/s1, Supplementary Material 1: Figure S1. Quality control of transcriptome and lipidome in Tan lambs. Figure S2. Heatmap generated from differentially expressed genes (DEGs) from different compared groups using the hierarchical clustering. Figure S3. Correlation coefficients between lipid metabolites and differentially expressed genes (DEGs) in Tan lambs. Supplementary Material 2: Table S1. Summary of transcriptome sequencing quality. Supplementary material 3: Table S2. Kyoto Encyclopedia of Genes and Genomes (KEGG) pathways and Gene Ontology (GO) terms significantly enriched in the dorsal subcutaneous adipose tissue (DSAT) of Tan lambs.
