# Supplementary figures and images for: Supplementing Diets with Agriophyllum squarrosum Reduced Blood Lipids, Enhanced Immunity and Anti-Inflammatory Capacities, and Mediated Lipid Metabolism in Tan Lambs
Source: Animals (Basel). 2022 Dec 9;12(24):3486. doi: 10.3390/ani12243486 (PMC9774518; doi:10.3390/ani12243486)

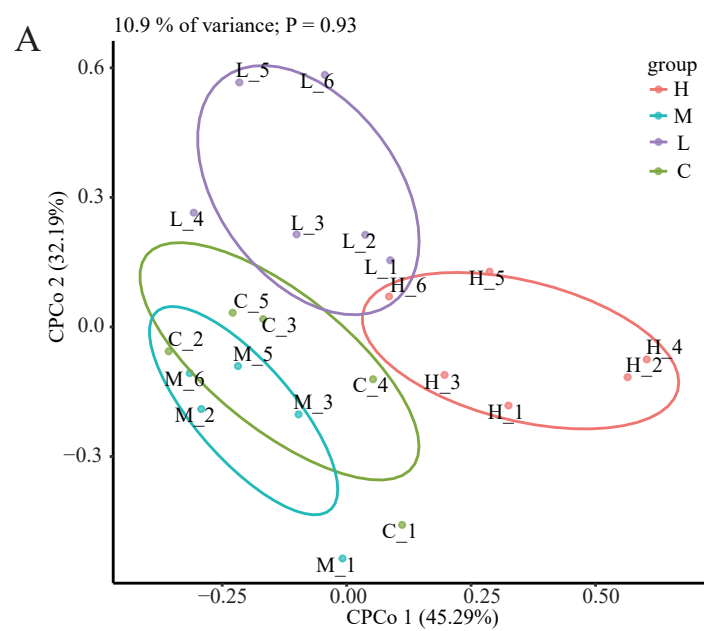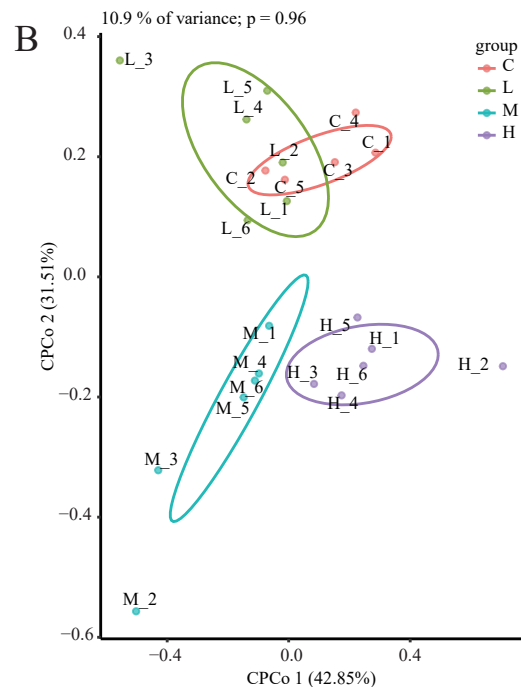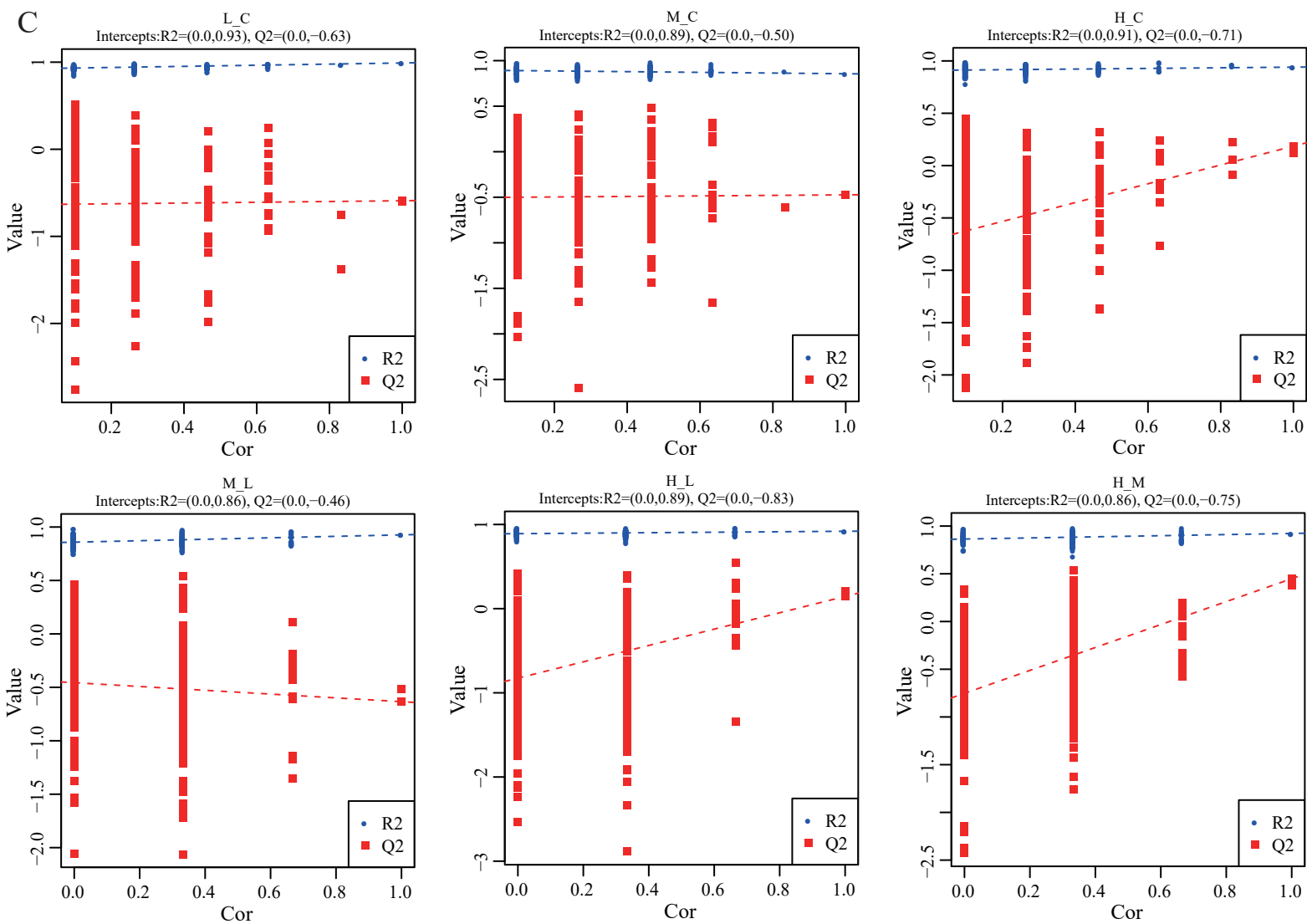

Supplement: Supplementary file 1 [file animals-12-03486-s001.zip › Figure S1.pdf]

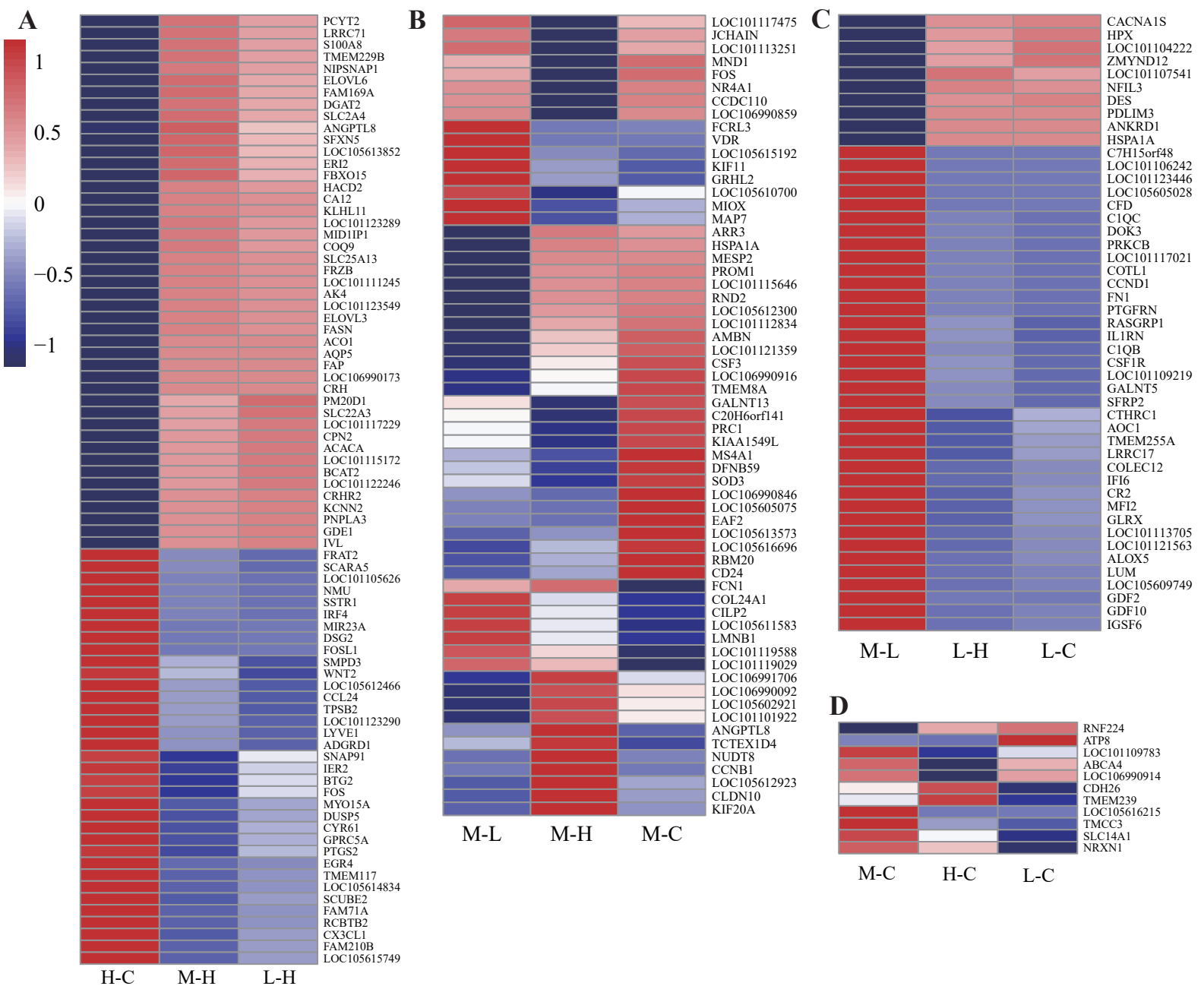

Supplement: Supplementary file 1 [file animals-12-03486-s001.zip › Figure S2.pdf]

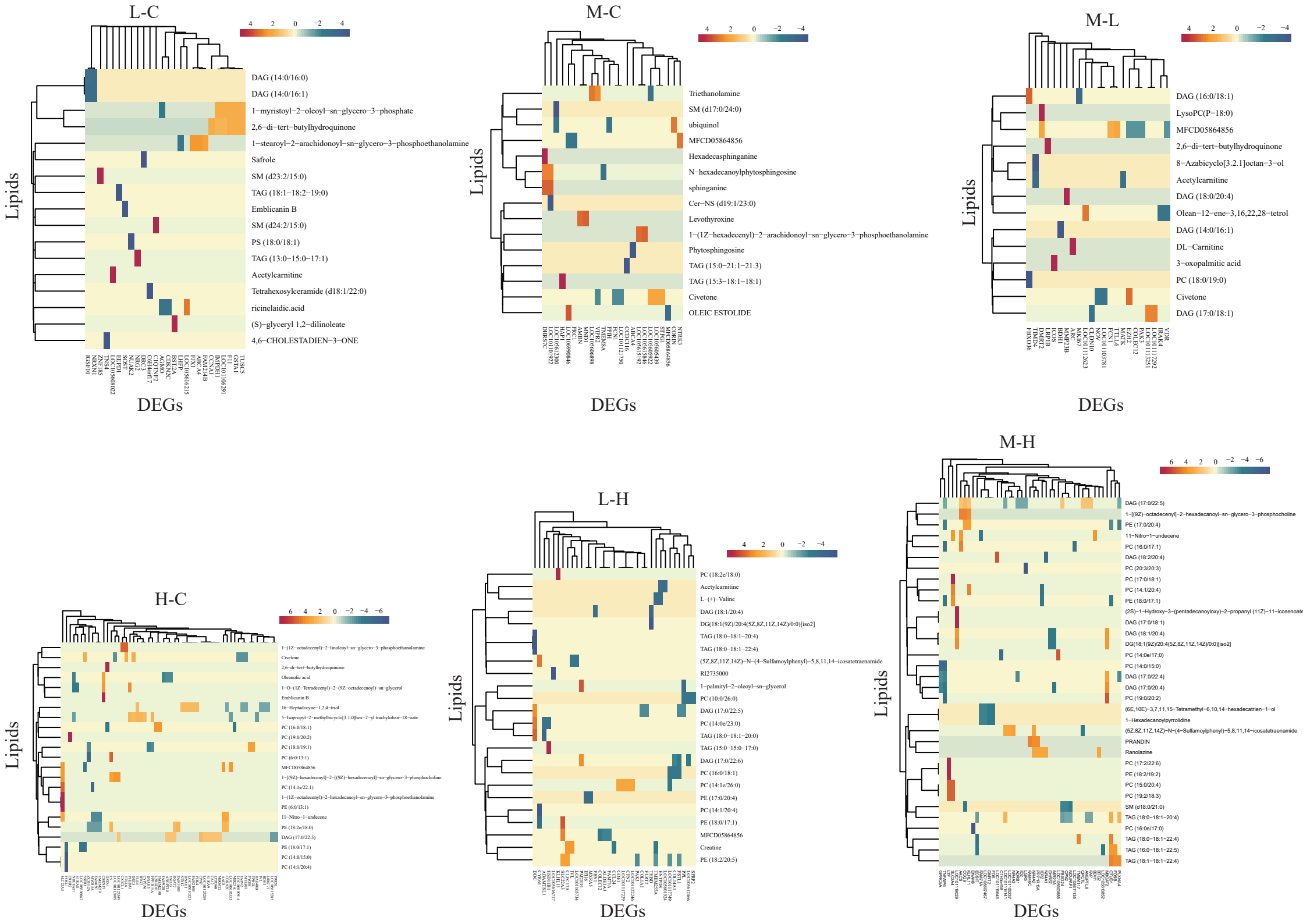

Supplement: Supplementary file 1 [file animals-12-03486-s001.zip › Figure S3.pdf]
